# Letter to the Editor: Interobserver Variability of Heart-to-Mediastinum Ratio in I-123 MIBG Sympathetic Imaging

**DOI:** 10.1007/s11886-012-0276-8

**Published:** 2012-05-09

**Authors:** Ben F. Bulten, Roel L. F. van der Palen, Hanneke W. M. van Laarhoven, Livia Kapusta, Annelies M. C. Mavinkurve-Groothuis, Lioe-Fee de Geus-Oei

**Affiliations:** 1 Department of Nuclear Medicine (757), Radboud University Nijmegen Medical Centre, Nijmegen, The Netherlands; 2 Department of Pediatric Hematology and Oncology, Radboud University Nijmegen Medical Centre, Nijmegen, The Netherlands; 3 Department of Medical Oncology, Radboud University Nijmegen Medical Centre, Nijmegen, The Netherlands; 4 Department of Medical Oncology, Academic Medical Centre, Amsterdam, The Netherlands; 5 Department of Pediatric Cardiology, Radboud University Nijmegen Medical Centre, Nijmegen, The Netherlands

To the Editor:

With great interest we read the article by Chen et al. regarding factors that affect heart-to-mediastinal ratio (H/M ratio) in I-123 meta-iodobenzylguanidine (MIBG) cardiac sympathetic imaging [1]. The authors describe the variation of H/M ratio among institutions and the factors that contribute to this. Furthermore, they provide different protocols that could minimize this variation.

One of the significant contributors to the variation of H/M ratio in cardiac imaging, are the settings of the regions of interest (ROIs) that are used. Currently, as correctly described by Chen et al. [1, 2], there are three main approaches for drawing the heart ROI. One is to place the ROI over the whole heart, thereby neglecting the “dilution” effect in patients with a dilated heart (ie, lower blood pool activity in the cavity than the myocardium) but resulting in high reproducibility [1, 3]. The second approach is to place the ROI along the left ventricular (LV) wall, inter alia described by Gerson et al. [4], which possibly leads to greater inter- and intraobserver bias [1]. In the third approach a smaller ROI is placed along the anterior part of the LV wall [2].

In our current ongoing study in pediatric patients by van der Palen et al.., we compared both approaches described by Chen et al. (ie, the “whole heart ROI” and the “small anterior wall ROI”). The reason for comparing those instead of the “LV wall ROI” by Gerson et al. is twofold: possible comparison of our results with Chen’s references and potential inaccurate placement of an ROI along the complete LV wall in small pediatric hearts (ie, the child’s heart is often too small for exact placement over the LV wall). Several studies in pediatric cardiology also use this approach [5, 6].

When studying the subsequently calculated H/M ratios we noticed that the interobserver variability when using these methods of ROI placement was only minor, when H/M ratios were obtained independently by two observers at 4 and 24 h post injection in our population of 30 pediatric patients. Intraclass correlation coefficients (ICCs) were as follows: for the small anterior wall ROI H/M ratio 0.882 (95 % CI: 0.578–0.956) and for the whole heart ROI H/M ratio 0.955 (95 % CI: 0.904–0.979) at 4 h. At 24 h, the ICC was 0.911 (95 % CI: 0.716–0.965) for the small anterior wall ROI H/M ratio and 0.951 (95 % CI: 0.896–0.977) for the whole heart ROI H/M ratio. Therefore, it can be concluded that the use of both ROI definition methods in children results in substantial to almost perfect interobserver agreement concerning H/M ratio. Furthermore, this suggests that whole H/M ratio has better agreement than small H/M ratio for both 4 and 24 h post injection scans.

Besides, both methods also showed a high within-patient ICC ratio of 0.865. This implies that intrapatient variability is fairly small and both methods give almost identical results.

Although the ICCs of both methods of ROI placement are rather similar, the most promising method is the whole H/M ratio 4 h post injection. To reduce differences in H/M ratios due to interobserver variability the method of ROI placement should be standardized, and it is reasonable to define this method as the gold standard for H/M ratio measurement.

However, as stated in the article by Chen et al. [1], this method could underestimate the H/M ratio in patients with a dilated heart (being the target population in the article by Chen et al.) and a relatively high negative contribution of the myocardial cavity. This, however, has no impact on our population, which does not contain patients with cardiomyopathy. Small anterior wall H/M ratios do not have this disadvantage and showed adequate intraobserver agreement.

Therefore, we can conclude that the best method to determinate the H/M ratio in cardiac sympathetic imaging depends on the patient characteristics of the study group. In pediatric patients we recommend the use of the whole heart H/M ratio, since it shows the best intraobserver agreement. With Chen we would like to emphasize the importance to standardize ROI placement methods to compare study results and minimize the intrapatient, interpatient, and interinstitutional variation.
